# Reply to the *Dental Digest*

**Published:** 1900-04

**Authors:** C. W. F. Holbrook

**Affiliations:** Newark, N. J.


### Domestic Correspondence.

### REPLY TO THE "DENTAL DIGEST."

To the Editor:

Sir,—There appeared in the December *Digest* an article entitled "Are there Traitors in the Camp?" the writer of which, after referring by name to certain Newark dentists who had made an honorable settlement with the Crown Company, says as follows:

"Personally, we believe this so-called settlement is merely an arrangement between these men and the 'Crown Company,' the ulterior purpose of which is to make it appear that some members at least have no confidence in the Protective Association, as this would, of course, tend to weaken the influence of our organization," etc.

During the Dental Convention at Niagara Falls, when the newspapers published the decree of the court in favor of the Tooth Crown Company, Dr. Crouse declared that he was utterly surprised and dumbfounded, as he even did not know the suit was pending. All who were present at the Convention remember his talk, and that he sent for his lawyer, who also expressed surprise and professed ignorance of the whole matter. At that moment the idea occurred to me that if the men to whom we paid money to protect our interests could not keep track of what was going on in open court in a suit the outcome of which would be of more vital importance to the dental profession than any ever before instituted, then I need no longer look to Dr. Crouse or his lawyer for protection. That view of the matter would, naturally, destroy confidence. But equally bad, or perhaps worse, is the only alternative view,—that these two men, Dr. Crouse and his lawyer, were cognizant of the suit. And now, as if to further test our credulity, they cry "Traitor" when a dentist complies with a ruling of the United States Court.

I am a member of the Dental Protective Association, and gave my money that I might be protected against fraud and imposture, but not for witnesses like those described in Judge Townsend's opinion:

"To further support the defence of anticipation, *the defendant has introduced the same witnesses who, and the same exhibits which, were before the court in the Bennett case. The inexplicable contrast between the statements of the same persons in the two cases is either an object-lesson as to the fallibility of human memory and the uncertainty of human testimony, or is forcibly suggestive of perjury and fraud. . . .*

"*In the present suit not one of these witnesses is able positively to identify said exhibit,* and the 'wife of a clergyman and her two daughters' *now testify, after an examination of church records, etc., that they were mistaken in their former testimony,* and that the cap was not put in Mrs. Martz's mouth until 1878, or until after the Low invention was completed, as found in the Richmond case and further proved herein. Even Dr. Beardslee now says that he cannot now testify that said work was done any earlier than the year 1878, and that, so far as he knows, the testimony of the Martzes as to the date when it was done is correct. *And further, as if to cap the climax of these contradictions, an apparently disinterested witness, Dr. Palmer, testified that he himself made the Beardslee-Martz exhibit,* and was told at the time that 'whatever of the kind I did was *for use in defending the suit* of the International Crown Company.' It is unnecessary to further discuss this branch of the case.

" . . . . *The defence of anticipation herein is overwhelmingly disproved by disinterested witnesses. The methods by which the Beardslee-Martz evidence of anticipation was secured by Dr. Beardslee in the Richmond case appears to have been questionable and reckless, and it is to be hoped that such practices are unusual. The contradictions in his testimony are so direct and material as to disentitle him to any consideration.* . . .

" Day's testimony has not been discussed because his veracity is attacked; his testimony is contradicted, and the facts stated by him, if true, would be insufficient for various reasons."

A few years ago this class of work was unknown to me, but there are others who claimed it was old. So we banded together to raise a fund that might be used to investigate and find out the former state of the art, and Dr. Crouse was intrusted with the task and the funds. What he has done is here stated by Judge Townsend in the United States Court. How he has spent the funds we do not know, but we think if he has proper evidence he must produce it soon or be branded as he deserves.

If the *Digest* is the mouthpiece of the dental profession, why has not the decree of the court appeared in it? Is the profession not capable of reading it understandingly?

Now, these are some of the reasons why we paid the Crown Company.

C. W. F. HOLBROOK.

NEWARK, N. J.

[The foregoing communication is published that both sides of this controversy may be heard. Some portions of the original communication have been omitted as irrelevant to the main issue.—ED.]

